# Public Health Strategies in the Face of Crisis: A Comprehensive Review of the Zika Outbreak in India

**DOI:** 10.7759/cureus.58621

**Published:** 2024-04-20

**Authors:** Vaibhav Chandra, Abhay Gaidhane, Sonali G Choudhari

**Affiliations:** 1 Community Medicine, Jawaharlal Nehru Medical College, Datta Meghe Institute of Higher Education and Research, Wardha, IND

**Keywords:** preparedness, india, crisis management, infectious diseases, public health strategies, zika outbreak

## Abstract

This review provides a comprehensive analysis of the public health strategies employed during the Zika outbreak in India, focusing on the identification, surveillance, and containment efforts. The multifaceted approach, including vector control measures, healthcare infrastructure enhancement, and public communication strategies, played a pivotal role in mitigating the impact of the virus. Government policies and international collaborations emerged as influential factors, underscoring the significance of a coordinated response to infectious disease crises. The study emphasizes the importance of ongoing vigilance and preparedness in public health systems, acknowledging the dynamic nature of emerging infectious diseases. The Zika outbreak in India serves as a valuable case study, offering insights into the strengths and weaknesses of crisis management responses. As the global community faces ongoing health challenges, the lessons learned from this review contribute to refining strategies, improving coordination, and fostering a proactive and resilient approach to safeguarding public health.

## Introduction and background

The Zika virus (ZIKV), a member of the Flaviviridae family, has emerged as a significant global health concern in recent years, primarily transmitted through the bite of *Aedes* mosquitoes. Notably, its association with severe birth defects, particularly microcephaly, has prompted widespread attention and necessitated comprehensive public health responses. Understanding the virology, transmission dynamics, and clinical manifestations of Zika is crucial for developing effective strategies to mitigate its impact [1]. In crises such as the rapid spread of emerging viruses like Zika, the effectiveness of public health measures becomes paramount. These strategies encompass a range of interventions, from early detection and surveillance to vector control, healthcare infrastructure enhancement, and public communication. The ability to promptly implement and adapt these strategies is essential in safeguarding communities and minimizing the public health burden associated with infectious diseases [2].

The Zika outbreak in India presented unique challenges due to the country's diverse geography, population density, and pre-existing health infrastructure. The spread of the virus raised concerns about the immediate health risks and the potential long-term consequences, including economic and social implications. Examining the specific context of the Zika outbreak in India allows for a nuanced understanding of the complexities involved in managing such crises within varying regional and cultural settings [3]. This review aims to critically analyze the multifaceted public health strategies deployed during the Zika outbreak in India, evaluating their effectiveness and drawing lessons for future infectious disease management. Given the global rise in vector-borne diseases, understanding these strategies' successes and limitations is crucial for global health security. By delving into the multifaceted aspects of the crisis, from the initial identification of cases to the long-term consequences, this review seeks to offer insights into the effectiveness of different interventions. Furthermore, it aims to extract valuable lessons from the Indian response to inform future public health efforts in the face of emerging infectious diseases. Through a detailed examination of the strategies implemented, challenges faced, and outcomes observed, this review aims to contribute to the broader understanding of crisis management in public health.

## Review

Background

Historical Context of ZIKV Outbreaks Worldwide

The ZIKV was initially identified in 1947 in Ugandan monkeys and subsequently recognized in humans in 1952 [4]. Since then, Zika outbreaks have been documented in tropical Africa, Southeast Asia, and the Pacific Islands [5]. Before 2007, at least 14 documented cases of Zika existed, although it is likely that additional cases occurred but went unreported [5]. The first notable outbreak of Zika-induced disease was reported on the Island of Yap in the Federated States of Micronesia in 2007 [4]. In recent years, the ZIKV has rapidly spread globally, with confirmed transmissions reported in over 72 countries and territories [6]. Notably, the virus has been associated with a significant correlation between infection and microcephaly, a condition marked by abnormal infant brain development [6]. This swift geographic dissemination of the ZIKV aligns with a pattern intensified by the rapid and expansive human movement facilitated by modern transportation, urbanization, deficient water management, and inadequate vector control. The proliferation is further facilitated by large populations with limited immunity and the global spread of the *Aedes aegypti* mosquito [7]. Noteworthy, Zika outbreaks have occurred in various countries, including India [8]. The public health challenges these outbreaks pose underscore the imperative to fortify surveillance systems and implement effective prevention and control strategies to combat the Zika epidemic [6].

Introduction of ZIKV in India

The ZIKV was initially identified in East Africa in 1947 by the Rockefeller Foundation during investigations into the ecology of yellow fever [7]. Despite its subsequent identification with widespread distribution in Africa and Asia, it was not recognized for causing epidemics until 2007 [1]. The virus has since exhibited a concerning global spread, with documented transmission reported in over 72 countries and territories [9]. In India, the inaugural case of Zika was reported in 2017, followed by the confirmation of 157 laboratory cases of ZIKV, including 63 cases involving pregnant women in Rajasthan [8]. Although several Zika cases have been reported in India, there has been no isolation of the virus to date [10]. The current situation suggests that the virus in India is distinct from both African and Asian prototype strains, displaying a notable difference in replication patterns compared to the well-documented profuse replication of African and Asian prototype strains [10].

Epidemiological Overview of the Zika Outbreak in India

The ZIKV made its first appearance in India in 2017, with a notable incidence of 157 laboratory-confirmed cases, including 63 cases involving pregnant women, reported in Rajasthan [8]. Furthermore, in 2022, the virus was identified in multiple states across India, indicating its expanding geographical presence and underscoring the imperative to enhance surveillance efforts [11]. The virus detection in India raises significant concerns, particularly due to the *Aedes aegypti* mosquito, a well-established vector for ZIKV transmission [10]. In addition, historical data dating back to 1954 suggest that the ZIKV may not have been recently introduced to India, as the National Institute of Virology had previously identified ZIKV antibodies in samples from the Bharuch district [10]. These findings emphasize the critical need for sustained vigilance and preparedness within the public healthcare system to effectively monitor and respond to the ZIKV outbreak in India [8].

Identification and surveillance

Early Detection and Diagnosis of Zika Cases

Timely detection and accurate diagnosis of Zika cases are pivotal for effectively managing outbreaks. In India, the initial Zika case was documented in 2017, leading to subsequent reports of 157 laboratory-confirmed cases of the ZIKV, including 63 cases involving pregnant women in Rajasthan [8]. However, the virus has not yet been isolated [8]. Recognizing the potential threat, there is an urgent call to fortify India's public healthcare system in anticipation of a ZIKV outbreak, emphasizing the critical need for enhanced surveillance [8]. Concurrently, ongoing surveillance of ZIKV and Dengue viruses in Aedes mosquitoes collected in the field is underway in India to assess the potential risk of transmission [12]. Prevention and control strategies, including travel precautions, vigilance against mosquito bites, measures to minimize the risk of sexual transmission, and prompt medical attention for individuals experiencing acute illness with rash or fever, are strongly recommended [6].

Surveillance Systems in Place During the Outbreak

ZIKV surveillance in India commenced in March 2016, initially involving 10 laboratories, and was subsequently expanded to include 35 laboratories by mid-2017 [8]. However, a recognized imperative is establishing a structured Zika surveillance framework in India to systematically enhance the country's ability to monitor and respond to potential outbreaks [13]. Concurrently, surveillance of ZIKV and dengue virus in *Aedes* mosquitoes collected from the field is underway in India to evaluate and mitigate potential transmission risks [12]. The World Health Organization (WHO) is crucial in supporting countries in conducting surveillance and implementing control measures for arboviruses, as outlined in the Zika Strategic Response Plan [14]. Recommended prevention and control strategies include exercising caution during travel, avoiding mosquito bites, implementing precautions to minimize the risk of sexual transmission, and seeking prompt medical care for individuals experiencing acute illness characterized by rash or fever [6,14].

Challenges and Successes in Identifying and Monitoring Zika Cases

The identification and monitoring of Zika cases pose several challenges, encompassing the necessity for accurate diagnostic testing, the intricacies of clinical diagnosis due to similarities with other acute arboviral fevers, and the difficulty in swiftly identifying subclinical and viremic individuals [15,16]. Diagnosing ZIKV infections has progressively relied on nucleic acid tests, primarily due to the complexities of cross-reactivity with other flaviviruses encountered in serological tests [15]. Acknowledging these challenges, the public healthcare system in India has recognized the imperative of introducing a structured Zika surveillance system to effectively monitor and respond to potential outbreaks, particularly in the context of the country's favorable climatic conditions supporting year-round virus transmission [8,13]. A comprehensive approach involving enhanced surveillance, formulating clinical management guidelines, and reinforcing environmental surveillance and vector control measures is crucial for addressing the complexities of identifying and monitoring Zika cases [17].

Vector control measures

Overview of Aedes Mosquitoes as Vectors

Integrated vector control (IVC) represents a comprehensive strategy combining source reduction, larviciding, and mass trapping with non-insecticidal sticky traps to diminish mosquito populations in targeted areas [18]. The focus of vector surveillance and control should prioritize mosquito species capable of transmitting viruses, particularly *Aedes aegypti* and *Aedes albopictus*, with control activities generally applicable to both species [19]. Recognizing *Aedes *mosquitoes' diverse utilization of artificial and naturally confined larval habitats, effective habitat management becomes crucial, with control efforts concentrating on the most prolific and epidemiologically significant habitats [20]. While chemical interventions, such as insecticides like dichlorodiphenyltrichloroethane (DDT), have shown efficacy in vector control [21], non-chemical larviciding strategies, including larvivorous fish, oil coating, and mass trapping of larvae, offer alternative approaches to regulate mosquito populations [21]. Population reduction initiatives seek to minimize vector densities where feasible, while community involvement is deemed essential for supporting government and public health programs in mitigating mosquito populations [18]. *Wolbachia *infection introduces a novel approach utilizing *Wolbachia* bacteria-infected males of *Aedes albopictus*, released in specific areas to reduce the local population of this mosquito species and protect targeted households [18]. Changes in human habits, such as installing mosquito screening on windows and doors and using mosquito nets while sleeping during the daytime, are effective in reducing the risk of mosquito-borne diseases [20]. In addition, street cleansing aims to eliminate water-bearing containers and maintain clean drains through a reliable and regular system, preventing stagnation and mosquito breeding [20]. Despite the deployment of these vector control measures, critical analysis reveals implementation challenges. Logistical delays in the procurement and distribution of vector control supplies, as well as disparities in healthcare access, have impacted their overall efficacy. Particularly, vector control in rural areas has faced significant obstacles due to limited local healthcare infrastructure, highlighting the need for tailored approaches to address specific geographical and demographic contexts.

Implementation of Mosquito Control Strategies

Clean and drain standing water: Regularly cleaning and draining standing water sources is fundamental to mosquito control. Mosquitoes breed in stagnant water, and eliminating these breeding sites disrupts their life cycle. By consistently removing standing water from containers, gutters, and other potential habitats, individuals can significantly reduce the local mosquito population and the risk of mosquito-borne diseases [22].

Installing screens: Installing screens on windows and doors is a proactive and long-term solution for mosquito control. Screens act as physical barriers, preventing mosquitoes from entering homes and establishments. This measure protects against mosquito bites and enhances the overall indoor living environment. Installing screens is a practical and sustainable strategy to create a mosquito-free zone within living spaces [22].

Biological control methods: Employing biological control methods offers an eco-friendly approach to managing mosquito populations. This can involve introducing natural predators, such as certain species of fish or bacteria, to target mosquito larvae and reduce their numbers. Biological control methods aim to disrupt the mosquito life cycle without chemical interventions, contributing to sustainable and environmentally conscious mosquito control practices [22].

Community engagement: Encouraging local communities to participate actively in mosquito control programs can yield highly effective results. Community engagement initiatives may include organizing clean-up drives to eliminate potential breeding sites, monitoring standing water sources in public spaces, and ensuring proper waste disposal practices. By fostering a sense of ownership and responsibility, communities become integral partners in maintaining a mosquito-free environment [22].

Use of mosquito nets: Mosquito nets protect against mosquito bites, particularly during sleep. Mosquito nets, when properly treated with insecticides, can offer an additional layer of defense, reducing the risk of disease transmission. This strategy is especially important in regions with prevalent mosquito-borne diseases, providing individuals with a practical and affordable means of safeguarding their health [22].

Proper water management: Implementing proper water management practices, such as covering water storage containers and eliminating standing water in flowerpots, is crucial in diminishing mosquito breeding sites. By addressing these potential habitats, communities contribute to reducing mosquito populations, thereby curbing the prevalence of mosquito-borne diseases [22].

Application of mosquito repellent sprays: The application of mosquito repellent sprays serves as a proactive and personal protective measure to reduce mosquito populations and shield individuals from mosquito bites. This approach is particularly valuable in regions where mosquitoes are prolific, and the risk of disease transmission is high. Regular use of repellent sprays contributes to individual health protection and overall community well-being [22].

Innovative technologies: Embracing innovative technologies founded on scientific research and validated methods represents a transformative approach to mosquito control. By adopting cutting-edge technologies, India can break away from historical stagnation in mosquito control efforts, ushering in a new era of resilience against mosquito-borne diseases. Integrating modern technologies offers promising solutions for more effective and sustainable mosquito control [23].

Integrated management of dengue: The integrated management of dengue involves the development of robust surveillance capabilities, integrating epidemiological and entomological data, and community engagement. This comprehensive strategy aims to implement effective measures to reduce the incidence of dengue. By combining data-driven approaches with community involvement, nations can proactively manage and control dengue outbreaks [24].

Use of novel technologies: Leveraging novel technologies, such as gene drives and paratransgenesis, for mosquito control and transmission prevention, represents an innovative frontier in disease management. These technologies offer advanced tools for improving disease surveillance and controlling mosquito-borne diseases. Integrating these novel approaches into existing strategies can enhance the overall effectiveness of disease prevention efforts [24].

Community Engagement and Awareness Programs

The first initiative, the engAGED Community Awareness Toolkit, is specifically crafted to assist organizations in heightening community awareness regarding the significance of social engagement for older adults [25]. This toolkit aims to provide valuable resources for organizations seeking to emphasize the importance of social connections for the well-being of older community members. Harvard University's Community Engagement Program constitutes the second endeavor, collaborating with researchers to enhance research projects by involving stakeholders with unique expertise and experience [26]. The program is dedicated to accelerating the adoption of evidence-based programs that prevent diseases and eliminate disparities, underscoring the role of community engagement in advancing public health initiatives.

**Figure 1:**
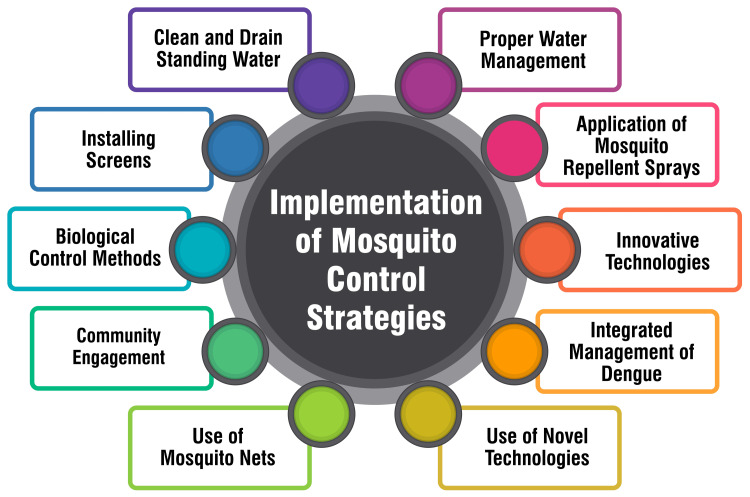
Implementation of mosquito control strategies Image credit: Dr. Vaibhav Chandra

In the realm of substance use prevention, the Substance Abuse and Mental Health Services Administration (SAMHSA) introduced a guide centered on leveraging community engagement to support substance use prevention [27]. This comprehensive guide systematically reviews the evidence related to community engagement, aiming to fortify the development of effective and equitable substance use prevention systems. The fourth initiative introduces the Community Engagement Framework, which operates based on principles that uphold the right of all community members to be informed, consulted, involved, and empowered [28]. As a strategic process, community engagement, as per this framework, is designed to collaborate with communities in identifying and addressing issues that impact their overall well-being. Lastly, universities play a significant role in community engagement, as demonstrated by programs like the University of San Diego's Community Engagement program, which partners with public and private sectors to address health concerns [29]. These university-driven initiatives highlight the potential of community engagement in addressing health issues and promoting public well-being. By involving communities in developing and implementing health programs, these initiatives can be tailored to meet specific community needs, thereby having a more profound impact on health outcomes. Figure 1 shows an implementation of mosquito control strategies.

Healthcare infrastructure and capacity building

Assessment of Existing Healthcare Facilities

Various programs and studies in India have consistently directed their attention toward evaluating existing healthcare facilities and enhancing the capabilities of health managers. Notably, in Tumkur, India, a capacity-building intervention was implemented to elevate the performance of local health services managers operating at the district level. This intervention aimed to furnish health services managers with essential public health management capacities, fostering an environment conducive to successfully decentralizing healthcare services [30]. Moreover, the National Urban Health Mission in India has introduced a "Capacity Development Framework" designed to fortify the implementation of urban health programs, focusing on addressing the needs of the urban poor and disadvantaged populations [31]. Concurrently, there is a renewed emphasis on achieving Universal Health Coverage (UHC) in India, with Health Technology Assessment (HTA) gaining recognition as a pivotal tool for advancing UHC goals. The development and application of HTA in India are considered indispensable for bolstering national capacity and expediting the nation's progress toward achieving UHC [32,33]. These comprehensive initiatives and frameworks underscore the ongoing commitment to evaluate and enhance healthcare infrastructure and capacity throughout India. They reflect a strategic and concerted effort to fortify the capabilities of health managers, particularly at the local and urban levels, while aligning with broader national goals of achieving UHC and employing sophisticated tools like Health Technology Assessment to guide decision-making processes in the healthcare sector [32,33].

Strengthening Healthcare Infrastructure for Zika Response

Prevention and control strategies: One key facet involves implementing proactive measures to mitigate ZIKV exposure. This encompasses avoiding travel to affected areas, taking precautions to minimize the risk of mosquito bites, adopting preventive measures against sexual transmission, and seeking prompt medical care for any acute illness featuring rash or fever [6].

Community initiatives: Another critical approach involves engaging communities in actively supporting local government and public health programs to reduce mosquito populations. Community participation includes covering water storage containers, eliminating standing water in flowerpots, and participating in clean-up activities to manage trash and used tires [14].

Laboratory surveillance: Establishing a network of laboratories, specifically Viral Research and Diagnostic Laboratories (VRDLs), is vital in enhancing ZIKV detection and diagnosis capabilities. This strategic step in laboratory surveillance contributes significantly to the preparedness for managing epidemics and natural calamities [14].

Healthcare infrastructure: Ensuring a robust healthcare infrastructure is paramount for effectively controlling ZIKV disease. This includes fortifying essential facilities, such as hospitals, educational institutions, non-governmental organizations (NGOs), community-based organizations, and the corporate sector. A well-established healthcare infrastructure is instrumental in mounting an efficient response to Zika outbreaks [34].

International collaboration: Coordinated international efforts play a pivotal role in minimizing the global threat and reducing the risk of a Zika epidemic. This involves the prompt and transparent reporting of the Public Health Emergency of International Concern (PHEIC) information to the WHO. Such collaboration facilitates a collective understanding of control measures and prioritizes ongoing research and development initiatives [34].

**Figure 2:**
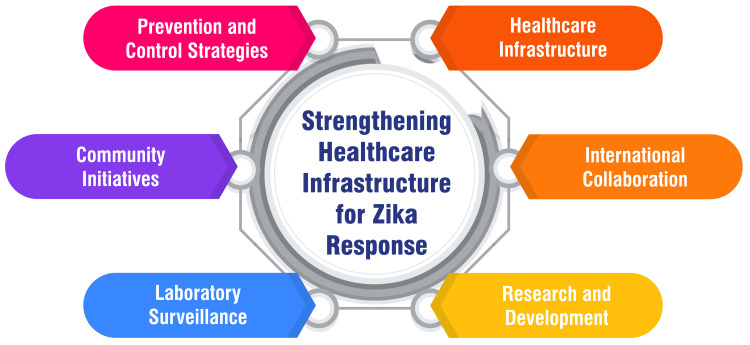
Strengthening healthcare infrastructure for Zika response Image credit: Dr. Vaibhav Chandra

Research and development: The intensification of research and development efforts for ZIKV diagnostics, vaccines, and therapeutics represents a proactive stance in addressing the virus comprehensively. Elevating these research and development endeavors is crucial for staying ahead of the curve in combating Zika outbreaks and minimizing the potential for transmission and associated complications [34]. Figure 2 shows the strengthening healthcare infrastructure for Zika response.

Training Healthcare Professionals for Effective Management

Improving laboratory capacity: The imperative to enhance the infrastructure and capacity of laboratories constitutes a foundational aspect in effectively combating the ZIKV and other infectious diseases. This enhancement is critical for ensuring that laboratories are well-equipped to conduct accurate and timely diagnostic testing. By fortifying laboratory capabilities, the healthcare system is better positioned to provide swift and precise diagnoses, enabling more effective disease management and public health responses [35].

Training healthcare professionals: A pivotal element in the comprehensive strategy involves the targeted training of healthcare professionals to manage and respond proficiently to ZIKV cases. This training encompasses educating healthcare workers on best practices for supporting affected individuals. Particularly crucial is disseminating knowledge about prevention and education strategies, with a specialized focus on key populations, such as pregnant women and their partners. This proactive approach enhances the healthcare workforce's preparedness and ensures the implementation of informed and effective measures to safeguard vulnerable populations [35].

Enhancing public health surveillance: Strengthening the public health surveillance system emerges as a vital component in the early detection and response to potential ZIKV outbreaks. This involves fortifying the capacity to systematically track the presence of the virus in communities and mosquitoes. A robust surveillance system facilitates the timely identification of potential outbreaks, allowing for swift and targeted intervention measures. This proactive monitoring contributes significantly to the overall effectiveness of public health responses [8].

Deploying rapid response teams: Establishing rapid response teams stands out as a proactive measure to curtail potential clusters of ZIKV cases. Rapid response teams are strategically positioned to facilitate swift and coordinated responses to emerging outbreaks. Their deployment is instrumental in containing the spread of the virus, minimizing the impact on affected populations, and ensuring a well-coordinated approach to outbreak management [35].

**Figure 3:**
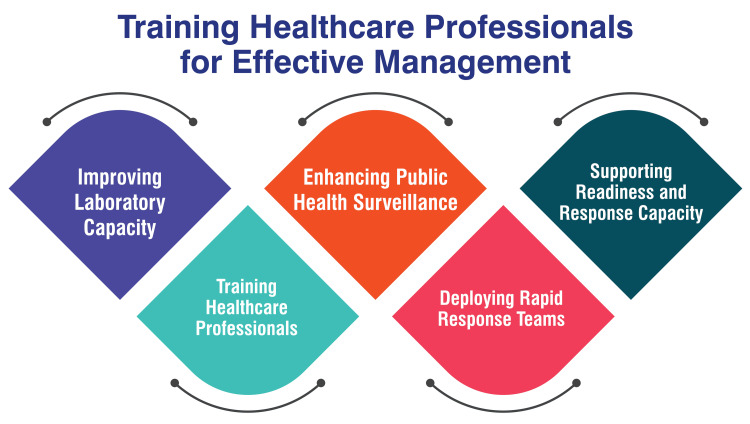
Training healthcare professionals for effective management Image credit: Dr. Vaibhav Chandra

Supporting readiness and response capacity: Providing comprehensive support for readiness and response capacity in affected areas, including the ongoing training of healthcare workers, constitutes a critical aspect of preparedness and mitigation efforts. This multifaceted support ensures that healthcare systems are well-prepared to address potential ZIKV outbreaks. By continually enhancing the readiness and response capabilities, communities are better equipped to manage and mitigate the impact of the virus on public health [35]. Figure 3 shows training healthcare professionals for effective management.

Public communication and education

Development of Communication Strategies

In public health, developing communication strategies is pivotal in educating and informing the public about the risks and prevention of diseases, such as the ZIKV. The Centers for Disease Control and Prevention (CDC) offers complimentary access to extensive courses, encompassing classroom training, webinars, and online self-study options, covering diverse public health and healthcare topics, including the ZIKV [14]. The ZIKV' prevalence across Latin America and parts of the United States in 2016 and 2017 serves as a valuable reference point for crafting communication plans tailored to future disease outbreaks. Public health communicators and practitioners employed various methodologies to effectively engage with the public, including fieldwork, community meetings, and interactions with healthcare providers [36].

Recognizing the influential role of social media, health promotion, communication, and education efforts during ZIKV outbreaks are increasingly conducted on this platform. Leveraging social media proves instrumental in educating the public on essential preventative measures, including the significance of barrier contraception, the proper use of mosquito repellents and nets, the adoption of protective clothing, and the advisability of avoiding travel to affected areas [37]. Equally critical is the preparedness of the public healthcare system for a potential ZIKV outbreak [8]. The Pan American Health Organization (PAHO) has taken a proactive stance by developing a comprehensive guidance resource package for country offices. This package includes coordinated planning, key messaging strategies, and recommended actions for risk communication and community engagement, all aimed at enhancing ZIKV prevention and control efforts [38].

Dissemination of Information to the Public

Effectively disseminating information to the public has emerged as a pivotal element in managing the Zika outbreak, constituting a critical facet of public health communication and education. Diverse communication strategies and platforms have been employed to relay preventative methods and risk awareness information. Traditional approaches, including community meetings and direct communication with healthcare providers, have been complemented using non-traditional channels, such as social media [36,37]. The CDC has played a crucial role in enhancing healthcare providers' preparedness through its CDC TRAIN program, offering comprehensive training courses. These courses, presented in various formats, such as classroom training, webinars, and online self-study options, cover a wide spectrum of public health and healthcare topics, including those relevant to the ZIKV [14].

Concurrently, concerted efforts have been made to fortify the public health surveillance system in anticipation of potential ZIKV outbreaks [8]. The WHO has contributed to this comprehensive approach by developing guidance and resource packages for risk communication and community engagement. Emphasizing the dynamic nature of the outbreak, these resources underscore the importance of adapting messages and actions based on the evolving epidemiology of the ZIKV [38]. These multifaceted efforts collectively reflect a comprehensive and adaptive approach to public communication and education throughout the Zika outbreak. By leveraging various strategies and channels, health authorities aim to ensure that accurate and timely information reaches the public, thereby enhancing preparedness, promoting preventative measures, and fostering a collective response to mitigate the impact of the ZIKV.

Addressing Misinformation and Promoting Preventive Measures

Throughout the Zika outbreak, a spectrum of risk communication strategies played a crucial role in preventing and controlling the spread of the virus. These encompassed diverse approaches, such as community-based participatory research, health fairs, health education delivered through theatrical means, and comprehensive social media campaigns [36,37,39]. Public health communicators and practitioners adopted a multifaceted approach, engaging in fieldwork, organizing community meetings, and establishing contact with healthcare providers to communicate critical information effectively [36]. Social media emerged as a particularly impactful platform for health promotion, communication, and education, offering an avenue to disseminate information on preventative methods during ZIKV outbreaks [37]. Recognizing social media's dynamic and pervasive nature, health authorities leveraged this channel to reach diverse audiences and amplify key health messages.

The Risk Communication and Community Engagement for Zika Virus Prevention and Control guidance resource package for country offices was developed to provide structured guidance. This resource package facilitated coordinated planning, outlined key messages, and detailed recommended actions for risk communication and community engagement for ZIKV prevention and control [38]. Concurrently, the preparedness of the public healthcare system for a ZIKV outbreak underwent evaluation, identifying opportunities to fortify the public health surveillance system [8]. This proactive assessment aimed to ensure a robust and responsive healthcare infrastructure better equipped to manage and mitigate the impact of potential ZIKV outbreaks through enhanced surveillance and communication strategies.

Government policies and coordination

Analysis of Government Policies During the Zika Outbreak

During the Zika outbreak, government policies and coordination were centred around a diverse set of strategies to prevent and control the spread of the virus. The United States Government's response plan strongly emphasized close coordination with state, local, tribal, and territorial governments. This comprehensive plan delineated key objectives, tasks, decision points, and the specific roles and responsibilities assigned to federal departments and agencies. The overarching goal was to facilitate a well-coordinated response to the outbreak, ensuring a cohesive and effective approach [40]. Aligned with global recommendations, prevention and control strategies advocated by the WHO and various research articles encompassed reducing exposure to the vector. This involved implementing measures like bed nets and mosquito repellents and environmental interventions like covering water storage containers and eliminating standing water to curtail mosquito populations. The WHO also underscored the importance of practicing safer sex or abstinence, particularly for individuals residing in regions with active transmission of the ZIKV [14]. Moreover, research findings highlighted the crucial role of community initiatives, including local mosquito control efforts and coordinated actions among neighbours, in enhancing the overall efficacy of Zika control [8,41]. These insights underscore the significance of adopting a multifaceted approach that integrates government coordination, community engagement, and individual-level preventive measures to effectively address the Zika outbreak's complexity. By combining these elements, authorities sought to create a comprehensive and synergistic strategy for managing the outbreak and safeguarding public health.

Inter-agency Coordination and Collaboration

Federal response: The United States Government has established a comprehensive ZIKV disease plan as a guiding framework. This plan meticulously delineates objectives, key tasks, operational response phases, triggers/decision points, and the specific federal roles and responsibilities in addressing the ZIKV. It is intricately aligned with overarching preparedness and response goals, including those outlined in the HHS Zika Virus Disease Domestic Preparedness and Response Goals and Objectives, the CDC Zika Virus Action Plan, and the CDC Interim Response Plan [40]. This ensures a cohesive and standardized approach at the federal level, providing a clear roadmap for the coordinated response to the Zika outbreak.

Interagency collaboration: Recognizing the complexity of public health investigations, successful management necessitates robust interagency collaboration. Various governmental and non-governmental organizations must work synergistically, involving effective communication, resource sharing, and coordinated efforts to ensure timely and comprehensive investigations [42]. This collaborative approach enhances the collective capacity to address multifaceted challenges and optimizes the allocation of resources for an efficient response.

Community involvement: Local mosquito control initiatives, integral to reducing the burden of Zika, gain heightened efficacy when coordinated across neighboring communities [41]. By fostering community involvement and coordination, these initiatives can address the shared challenges of the Zika outbreak. This communal approach optimizes mosquito control efforts' impact and promotes a sense of shared responsibility and awareness at the grassroots level.

Ongoing communication: A resilient pandemic response relies on sustained and effective interagency communication. This entails ongoing collaboration between agencies to ensure a harmonized and efficient response. Timely sharing of information, updates on the evolving situation, and coordinated decision-making processes contribute to a cohesive and dynamic approach to pandemic management [43].

**Figure 4:**
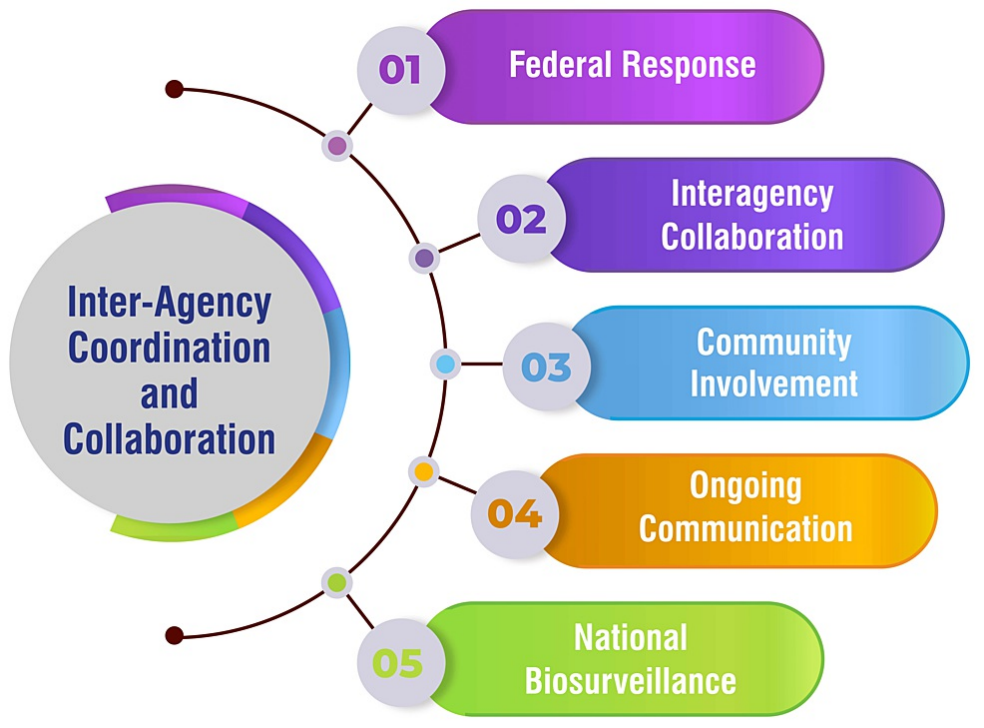
Inter-agency coordination and collaboration Image credit: Dr. Vaibhav Chandra

National biosurveillance: An integral component of a robust Zika response strategy involves the establishment of national biosurveillance capabilities. This entails fostering efficient interagency collaboration and coordination within federal agencies and extending to nonfederal entities. A well-structured national biosurveillance system enhances the capacity for early detection, swift response, and informed decision-making across agencies involved in addressing the Zika outbreak [44]. This ensures a comprehensive and proactive stance in managing the public health implications of the ZIKV. Figure 4 shows inter-agency coordination and collaboration.

Evaluation of Policy Effectiveness in Controlling the Outbreak

The efficacy of government policies in managing the Zika outbreak is intricately tied to the coordination and cooperation among diverse agencies and governmental levels. The Zika Virus Disease Contingency Response Plan established by the United States Government underscores the imperative of cohesive efforts involving federal, state, local, tribal, and territorial governments to effectively respond to the outbreak [40]. Key elements highlighted in this plan include interagency cooperation and continual communication, which are essential components for an efficient pandemic response [43]. The Government Accountability Office further underscores the significance of national biosurveillance and interagency collaboration as components of a robust Zika response strategy [45].

In the Indian context, the readiness of the public healthcare system for a potential ZIKV outbreak and identifying opportunities to fortify the public health surveillance system are deemed essential for an effective response and control [8]. In addition, a study underscores the positive impact of coordination among neighbors in enhancing the effectiveness of Zika control initiatives, emphasizing the crucial role of local cooperation and coordination [41]. Therefore, the assessment of policy effectiveness in managing the Zika outbreak underscores the critical importance of interagency collaboration, coordination across different levels of government, and the reinforcement of public health surveillance systems.

International collaboration and assistance

Involvement of International Organizations and Agencies

Global Virus Network (GVN): The GVN plays a pivotal role in the global response to the Zika epidemic through its dedicated Zika Task Force. This task force collaborates extensively with GVN's Centers of Excellence and other pertinent organizations to respond to the ZIKV. It actively forges strategic alliances and effectively communicates with various entities to identify opportunities for collaborative efforts [46]. This collaborative approach ensures a synergistic and coordinated response to the Zika epidemic on a global scale.

CDC: The CDC has emerged as a crucial entity in the worldwide response to Zika. The CDC collaborates with diverse partners, extending valuable assistance to public health organizations across different countries. A notable example is the CDC's support to national public health partners, assisting various jurisdictions in their Zika responses [47]. Through its expertise and collaborative initiatives, the CDC contributes significantly to the global efforts to address and mitigate the impact of the ZIKV.

WHO: The WHO actively participates in comprehensive initiatives, including the ZIKABRA study in Brazil, designed to enhance understanding of the ZIKV and inform preventive measures. Serving as a global health hub, the WHO mobilizes experts and resources to address Zika and tackle other pressing public health threats on a global scale [48]. The WHO's multifaceted involvement reflects its commitment to advancing knowledge, fostering collaboration, and coordinating responses to global health challenges.

USAID Global Health Supply Chain Program: The USAID Global Health Supply Chain Program has been instrumental in addressing emergent issues like Zika by facilitating global collaboration. The program actively promotes public health through regulatory support, contributing to developing and implementing effective strategies to manage and mitigate the impact of the ZIKV [49]. Its engagement underscores the importance of a well-coordinated and responsive global supply chain in addressing public health emergencies.

**Figure 5:**
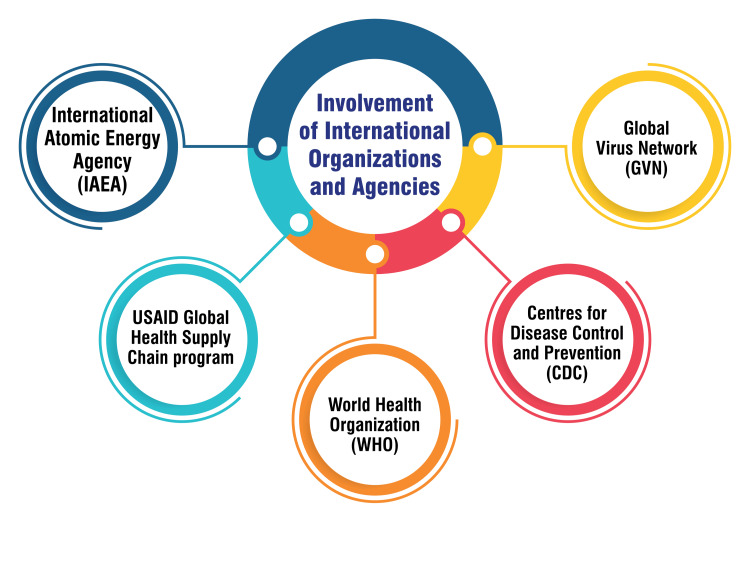
Involvement of international organizations and agencies Image credit: Dr. Vaibhav Chandra

International Atomic Energy Agency (IAEA): The IAEA, in collaboration with the Food and Agriculture Organization of the United Nations (FAO), actively supports its member states in detecting and controlling Zika outbreaks. This support is provided through technical cooperation and training initiatives, highlighting the role of expertise and knowledge-sharing in building the capacity of member states to manage and respond to Zika effectively [49]. The IAEA's engagement underscores the importance of a multidimensional approach, leveraging technical expertise to enhance the preparedness and response capabilities of nations facing Zika outbreaks. Figure 5 shows the involvement of international organizations and agencies.

## Conclusions

This comprehensive review of the Zika outbreak in India illuminates crucial strategies for effectively managing infectious disease crises. Early identification and surveillance efforts, robust vector control measures, and healthcare infrastructure enhancements played pivotal roles in containing the virus. Government policies and international collaborations underscored the importance of coordinated responses in crises. However, it is imperative to move beyond general conclusions and offer actionable recommendations. The review concludes that while India's response to the Zika outbreak involved comprehensive strategies, there is a need for enhanced preparedness, particularly in areas of rapid diagnostic testing, public health education, and inter-agency coordination. We recommend establishing a national infectious disease response framework, investing in community-based health education, and creating an inter-agency task force to ensure swift, coordinated action in future outbreaks. Furthermore, the findings highlight the necessity of ongoing vigilance and preparedness in public health systems, emphasizing the dynamic nature of infectious diseases and the need for adaptable strategies. The Zika outbreak in India serves as a compelling case study, emphasizing the importance of sustained investment in public health resources and infrastructure to ensure resilience in future health crises. As we reflect on the lessons learned, it becomes evident that a holistic and adaptive approach is essential for effective crisis management. Challenges such as socioeconomic impacts and healthcare disparities should guide future preparedness efforts, fostering a proactive and resilient global response to safeguard public health in an ever-evolving landscape of emerging infectious diseases.
